# Exploring the infiltrative and degradative ability of *Fusarium oxysporum* on polyethylene terephthalate (PET) using correlative microscopy and deep learning

**DOI:** 10.1038/s41598-023-50199-w

**Published:** 2023-12-27

**Authors:** Flavio Cognigni, Marta Elisabetta Eleonora Temporiti, Lidia Nicola, Nicolas Gueninchault, Solveig Tosi, Marco Rossi

**Affiliations:** 1 Department of Basic and Applied Sciences for Engineering (SBAI), University of Rome LA SAPIENZA, 00185 Rome, Italy; 2 Laboratory of Mycology, Department of Earth and Environmental Sciences, University of Pavia, 27100 Pavia, Italy; 3 Carl Zeiss X-ray Microscopy, Inc, 5300 Central Parkway, Dublin, CA 94568 USA; 4 National Biodiversity Future Centre, 90133 Palermo, Italy

**Keywords:** Fungi, Imaging techniques, Pollution remediation

## Abstract

Managing the worldwide steady increase in the production of plastic while mitigating the Earth’s global pollution is one of the greatest challenges nowadays. Fungi are often involved in biodegradation processes thanks to their ability to penetrate into substrates and release powerful catabolic exoenzymes. However, studying the interaction between fungi and plastic substrates is challenging due to the deep hyphal penetration, which hinders visualisation and evaluation of fungal activity. In this study, a multiscale and multimodal correlative microscopy workflow was employed to investigate the infiltrative and degradative ability of *Fusarium oxysporum* fungal strain on polyethylene terephthalate (PET) fragments. The use of non-destructive high-resolution 3D X-ray microscopy (XRM) coupled with a state-of-art Deep Learning (DL) reconstruction algorithm allowed optimal visualisation of the distribution of the fungus on the PET fragment. The fungus preferentially developed on the edges and corners of the fragment, where it was able to penetrate into the material through fractures. Additional analyses with scanning electron microscopy (SEM), Raman and energy dispersive X-ray spectroscopy (EDX) allowed the identification of the different phases detected by XRM. The correlative microscopy approach unlocked a more comprehensive understanding of the fungus-plastic interaction, including elemental information and polymeric composition.

## Introduction

Plastic is a fundamental part of everyday life, but also a serious problem plaguing our planet. The global plastic production dramatically increased from 2 Mt in 1950s to almost 390 Mt in 2021^1^. This increase, associated with the short lifespan of most plastic products and with its impacts on the ecosystems, contributes heavily to Earth global pollution^2^. Several studies explored possible strategies to mitigate this problem, focusing on the ability of microorganisms in bioremediation processes, which are defined as the employment of biological agents for the degradation and detoxification of hazardous materials or contaminants. These processes consist in a series of enzyme-catalysed physico-chemical transformations of the initial toxic substrate into simpler and less toxic units^3^. Fungi are ubiquitous microorganisms capable of forming vast mycelial networks, which allow their penetration into various recalcitrant substrates and the *in-situ* release of a wide range of powerful catabolic exoenzymes^4^. For these reasons, fungi are reported to be involved in biodegradation and bioremediation of different recalcitrant materials such as hydrocarbons, pesticides, and plastics^5^. Several studies report the involvement of fungi in plastic biodegradation thanks to the production of non-specific exoenzymes, able to break down different plastic polymers^6^. Nevertheless, the interaction of fungi with the plastic substrate is very difficult to study due to the deep hyphal penetration in plastic materials, which allows neither the visualisation of the fungus within the substrate nor its removal. These characteristics partially prevent a correct evaluation of the fungal plastic degradation, increasing the risk of misunderstanding the fungal activity.

Correlative microscopy refers to the integration of various investigative techniques to obtain a comprehensive understanding of the sample’s properties, encompassing its structural, morphological, compositional, and chemical attributes^7,8^. One of the driving tools of correlative microscopy is X-ray Microscopy (XRM), a non-destructive three-dimensional characterization method that harnesses the radiation-matter interaction^9,10^. The operational principles of XRM are based on micro–Computed Tomography (microCT) approach which involves the acquisition of a set of projections, i.e. radiographies, at various viewing angles by rotating the sample^11^. This technique unlocks the ability to identify distinct phases within the sample non-destructively. However, XRM enables sub-micron resolution analysis that cannot be achieved with microCT, thanks to its unique optics-based design. XRM offers a broad range of applications in energy materials research and batteries^12,13^, cultural heritage characterization and preservation^14–17^, biomedical engineering^18,19^, electronics and semiconductors^20,21^, life sciences^22–24^, and food analysis^25,26^. Deep Learning (DL) based XRM reconstruction is an emerging technology that involves the utilisation of trained neural networks following the acquisition of a set of X-ray projections (or radiographs) and the subsequent reconstruction of a volume. This approach offers significant benefits, such as the ability to effectively denoise (XRM) data and mitigate reconstruction-related artefacts, including aliasing artefacts (such as shadow bands, dark streaks, or noise-like distortions) that may arise when there is insufficient X-ray projection data available. While most machine learning applications in this field have primarily focused on post-reconstruction methods for tasks such as image segmentation, feature classification, and object recognition^27,28^, DL-based reconstruction represents a novel and promising technology in XRM research. We used a state-of-art DL reconstruction algorithm called *DeepScout* (Carl Zeiss X-ray Microscopy, Dublin, CA, USA)*,* which was applied here to Life Sciences research for the first time, to enhance XRM analysis^29^. The algorithm uses high-resolution XRM datasets as training data for lower resolution, larger Field of View (FOV) datasets and upscales the larger volume data using a neural network model. However, since XRM does not provide elemental/chemical information, other analyses such as scanning electron microscopy (SEM), energy dispersive X-ray spectroscopy (EDX) and Raman spectroscopy should be performed to reach a comprehensive understanding of the sample including its structural, morphological, compositional, and chemical properties.

In this study, we employed a multiscale correlative microscopy workflow to investigate the infiltrative capability and degradative effects of a fungal strain belonging to *Fusarium oxysporum* Schltdl*.* species on polyethylene terephthalate (PET). XRM coupled with *DeepScout* was used as a leading technique to observe the interaction between the fungal strain and PET. Additionally, SEM was used to investigate the fungal growth on the external surface of PET with higher resolution. EDX and Raman spectroscopy were used to determine the elemental composition and changes in the chemical structure of the material in relation to the presence of the fungus. This approach facilitated a comprehensive understanding of the three-dimensional morphology of the interaction, encompassing elemental information and polymeric composition.

## Results and discussion

### Identification of the fungal strain

The use of PET fragments as bait in Bosco Siro Negri soil plates allowed to isolate the strain F2 with high bioremediation potential against plastic, as demonstrated by the preliminary growth tests and qualitative enzymatic assays, which showed its ability to grow on PET and the abundant production of esterases and cutinases. The morpho-dimensional analysis, in combination with the molecular sequencing of the internal transcribed spacer fragment (ITS) of 18S rDNA, led to the identification of the fungal strain F2 as *Fusarium oxysporum* Schltdl. The abundant production of cutinases is a widely known characteristic of *Fusarium* species^30,31^, especially related to their plant pathogenic activities^32,33^. Indeed, cutinases are able to hydrolyse the ester bonds in cutin, a natural aliphatic polyester abundant in plant cuticle^34^, to simplify the fungal penetration in the host plants^33^. Thanks to these enzymatic characteristics and the presence of ester bonds in PET polymers, several studies were performed on the cutinases’ activity in PET degradation^35,36^. These studies reported the formation of PET monomer bis(2-hydroxyethyl) terephthalate (BHET) and monoester mono (2-hydroxyl ethyl)terephthalate (MHET) after the fungal cutinase action, highlighting the biodegradation potential of these enzymes. It was also supported by modification in water adsorption ability^37^ and in PET crystallinity^36^. Furthermore, cutinases of *F. oxysporum* were deeply investigated analysing temperature, pH and the presence of additives and surfactants to improve their involvement in PET degradation^38,39^. On the other hand, studies on the way strains of *Fusarium* penetrate into PET fragments are still lacking.

### Scanning electron microscope (SEM) analysis

**Figure 1:**
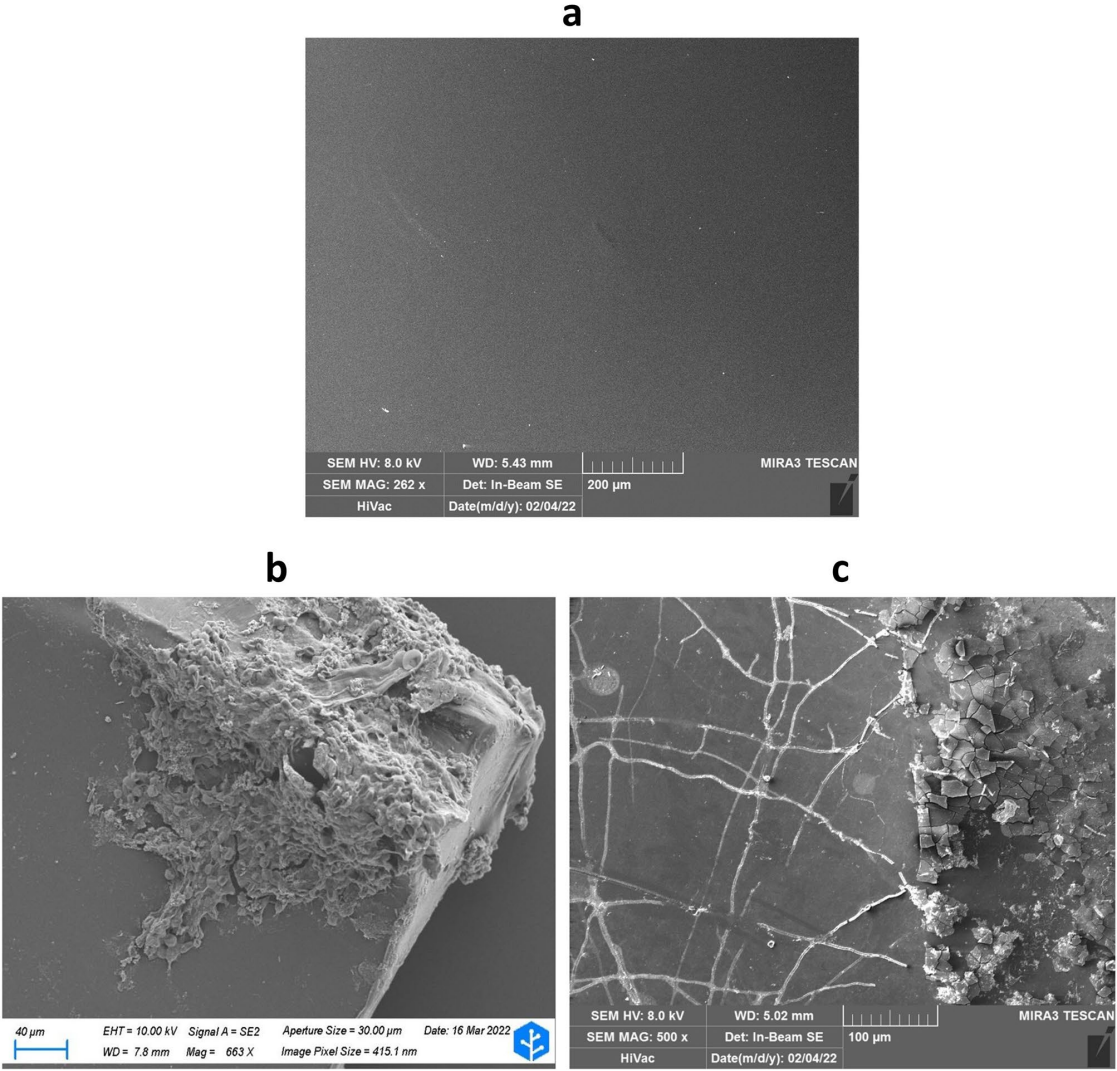
SEM imaging of PET reference sample and biodegraded fragment. SEM images showing (**a**) PET reference sample, (**b**) fungal growth on corners of PET fragment, and (**c**, left) fungal hyphae growing on the PET surface and (**c**, right) fragments of PET.

PET fragments colonised by F2, and reference PET fragments were observed using SEM to get a general idea of the appearance of the samples. The observation of the reference PET sample revealed a smooth and homogeneous surface (Fig. 1a). On the other hand, SEM analysis on the PET fragment attacked by F2 confirmed that a conspicuous fungal mycelium was visible on the corner of the fragment (Fig. 1b), and creation of cracks and micro fragments was observed on the PET surface in the proximity of fungal hyphae (Fig. 1c). According to the literature, the presence of cracks, holes, or fractures could be the indication of the biodegrading action of the fungus^40,41^.

### X-ray microscopy (XRM) analysis

**Figure 2:**
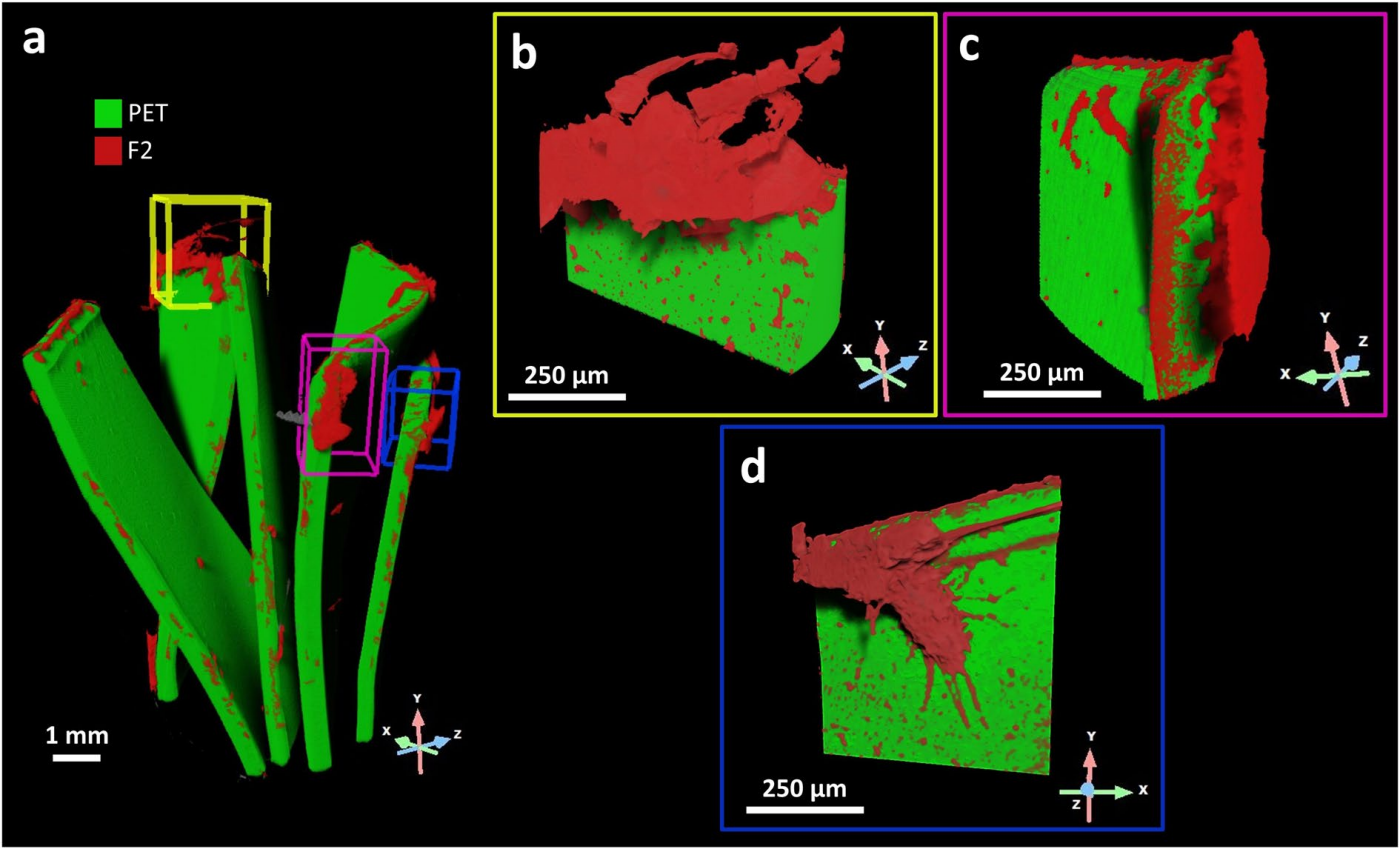
XRM 3D rendering of fungal colonisation on PET fragments. (**a**) XRM 3D rendering of several PET fragments with F2 *F. oxysporum* after 90 days of incubation. The fungus (red) covers 16% of the total available PET (green) surface and shows a preferential distribution along (**b**) edges (yellow box) and (**c**) corners (purple box) of PET fragments. However, F2 also attacked some (**d**) planar surface regions (blue box) of PET fragments.

The initial large-scale investigation was performed using XRM (pixel size 4.33 μm) to non-destructively investigate several PET fragments where the F2 was grown for 90 days, to detect large-scale features (Fig. 2a). The grey-scale level in a reconstructed XRM dataset represents the intensity of each pixel, indicating the amount of X-ray attenuation that occurred and enabling the segmentation of regions of interest (ROIs) through a histogram-based thresholding process. This method led to a three-dimensional reconstruction of the fungal growth and its distribution over the substrate (Fig. 2a).

Upon analysing the low-resolution dataset, we identified three distinct colonisation sites, namely the edge, corner, and planar surface of PET fragments that warranted further investigation at a higher resolution. The Scout & Zoom protocol was employed to identify a smaller volume of interest (VOI) that corresponded to the edge growth site. This selected VOI was then investigated via high-resolution XRM (pixel size 0.75 μm), aiming to delve into the intricate details of the fungal structure and its interaction with the substrate at smaller length scales (Fig. 2b). Furthermore, the high-resolution dataset obtained from the edge growth site was used as a training model for the *DeepScout* DL reconstruction algorithm. This approach facilitated the generation of higher-resolution images for the remaining VOIs under examination, i.e. corner, and planar surface growth sites. The strain F2, segmented in red, showed a preferential distribution along edges (yellow box, Fig. 2b) and corners (purple box, Fig. 2c) of PET fragments, segmented in green, covering 16% of the total available PET surface (Fig. 2). However, the fungal strain also attacked some planar surface regions (blue box, Fig. 2d) of PET fragments. Moreover, the fungus on PET did not appear to be a homogeneous phase. In fact, it was a multiphase region exhibiting variations in both morphology and composition. To gain a comprehensive understanding, we employed a multi-scale and multi-technique correlative microscopy approach. This involved the utilisation of micro-Raman spectroscopy, SEM, and EDX investigations, with the previous and subsequent XRM characterizations serving as guiding tools. The correlative microscopy workflow provided a method for the investigation of this complex system across dimensions and scales to investigate its physical, morphological, and chemical features^42^.

### Investigation of the different phases in the sample

**Figure 3:**
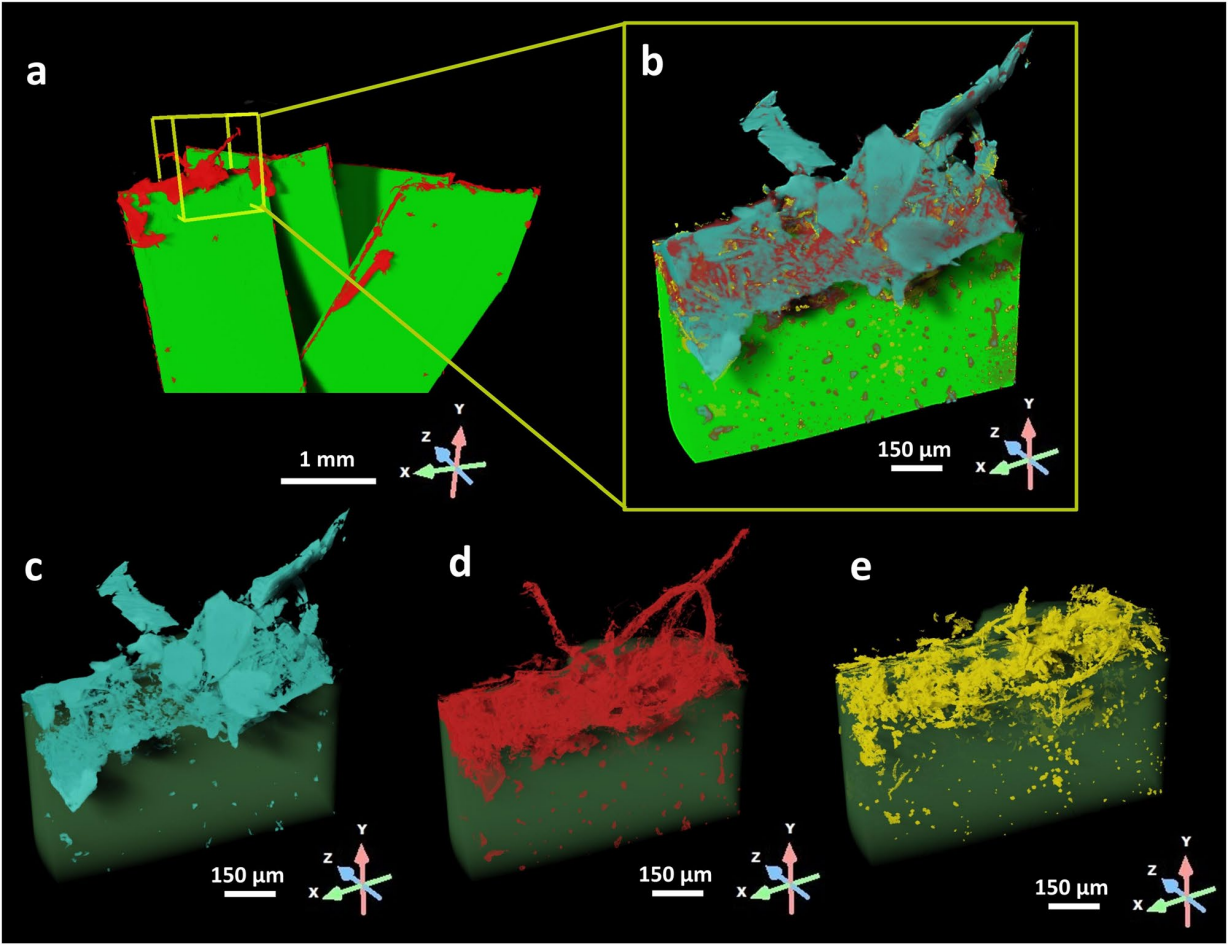
3D model reconstruction and phase segmentation. The high resolution XRM dataset corresponding to the edge colonisation growth site was further examined to investigate the composition of the sample. (**a**) 3D model of PET fragments (green) colonised by F2 *F. oxysporum* (red) where the yellow box highlights the VOI scanned using high-resolution XRM. (**b**) The VOI scanned in high resolution mode where three different phases were segmented: (**c**) salts crystals in light blue, (**d**) the fungus in red and (**e**) the biodegraded plastic due to the fungal strain attack in yellow.

According to their grey-scale level detected by XRM at the edge growth site (Figs. 2b and 3), three-different phases were detected (Fig. 3b): the light blue one showing mainly geometric shapes (Fig. 3c), the red one showing filaments (Fig. 3d), the yellow one without any defined shape (Fig. 3e). XRM allowed pinpoint precise micro-regions of the sample revealing their internal structure, morphology and phase composition without providing elemental/chemical information. Therefore, further investigation with complementary techniques in a correlative microscopy environment was required to enrich the global knowledge of the specimen.

**Figure 4:**
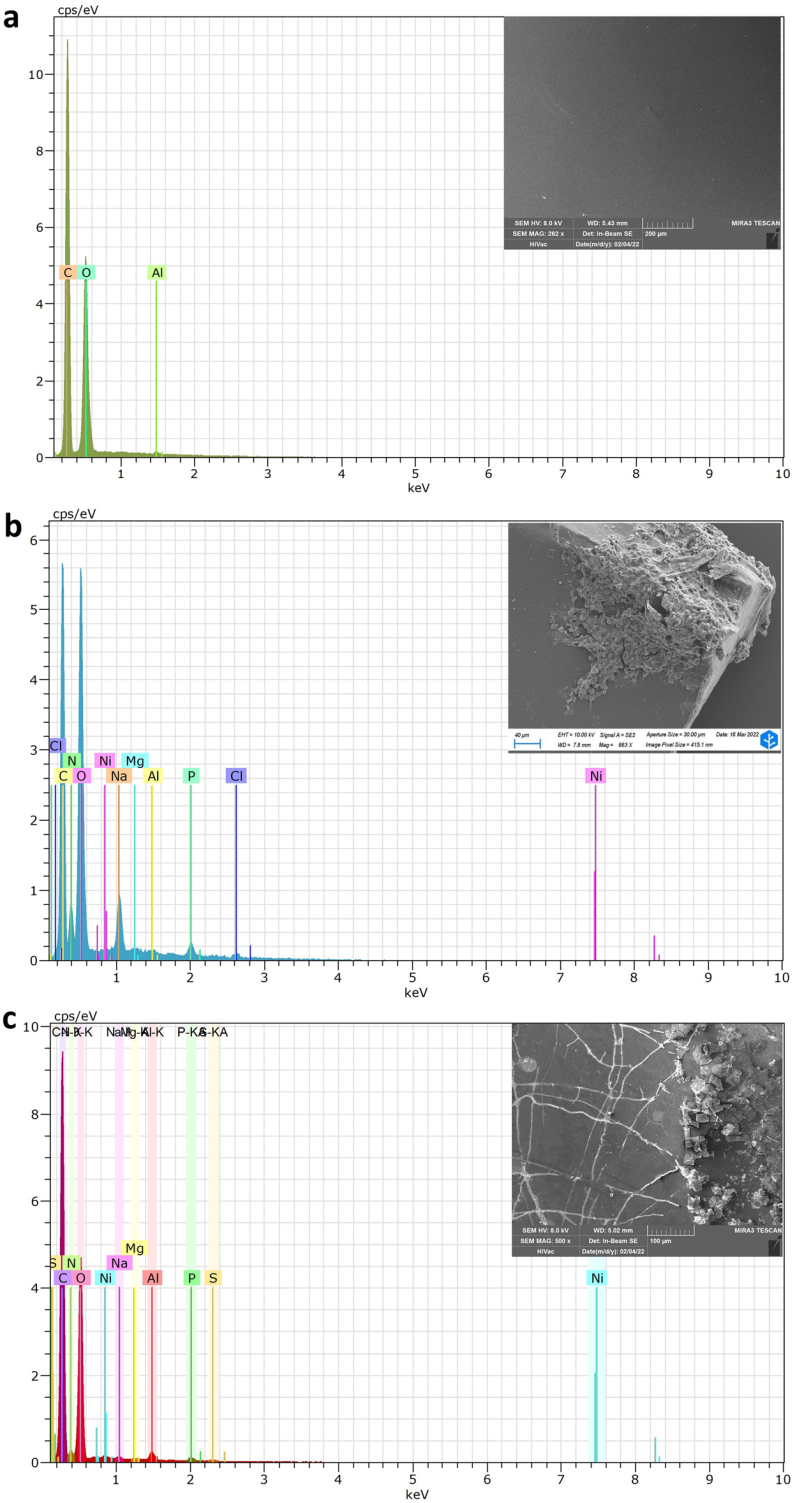
Energy dispersive X-ray (EDX) spectra. EDX spectra of (**a**) PET reference sample, (**b**) F2 *F. oxysporum* on PET fragment, and (**c**) PET fragment after F2 action.

Firstly, the PET fragments were examined with EDX for qualitative elemental determination. This analysis showed that PET reference samples were composed mainly of carbon (C) and oxygen (O), as might be expected from PET chemical structure (Fig. 4a)^43^. The presence of Aluminium (Al) was also detected, probably because this element is often used as a co-catalyst in the fabrication processes of PET^44^. When analysing the edge growth site with abundant F2 fungal hyphae, elements such as phosphorus (P), chloride (Cl), magnesium (Mg), sodium (Na), and nitrogen (N) were found (Fig. 4b). These elements can be labelled as elements composing the fungal cell and salt crystals of the cultural medium^45,46^. In fact, they are involved in numerous physiological and biochemical processes as well as in structural characteristics of the cell^47^. Nitrogen is often found bound to chitin in cell walls as amines and amides^48^, as well as being key components of amino acids^49^. Sodium and magnesium are involved in numerous enzyme activations and synthesis of nucleic acids and proteins^50^. When the PET surface with visible cracks and fractures was analysed, an expected predominance of carbon (C) and oxygen (O) was observed. However, small traces of the fungal characterising elements such as nitrogen (N), sodium (Na) and phosphorous (P) were detected, suggesting that the fungus released some biological molecules such as exoenzymes^38^ or micelles with anionic surfactant (e.g. sodium dodecyl sulphate), useful to facilitate the enzymes action (Fig. 4c)^51^_._

**Figure 5:**
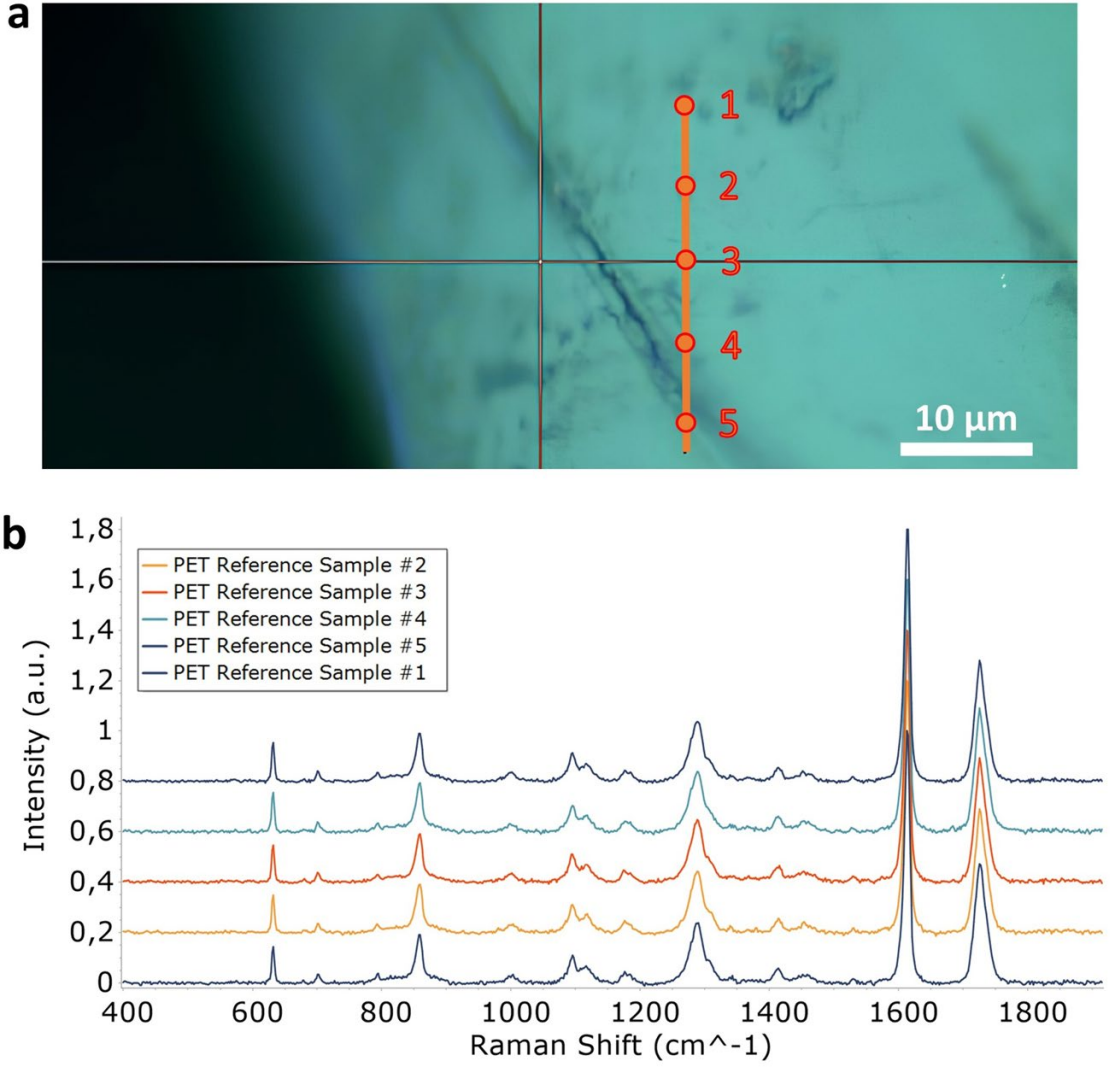
Raman spectra over a fracture on PET reference sample. (**a**) Spots analysed covering the fracture on the PET reference sample and (**b**) comparison between the spectra obtained from points 1 to 5.

Next, Raman spectroscopy was employed to investigate the effects of F2 biodegradative activity on the PET substrate using a Renishaw InVia Raman Microscope (Renishaw, Wotton-under-Edge, UK). Raman spectroscopy was first performed on five points on a fracture found on the PET reference sample (without F2, Fig. 5a). This analysis did not reveal any change in spectrum in any of the points (Fig. 5b), confirming the uniformity of the PET material.

**Figure 6:**
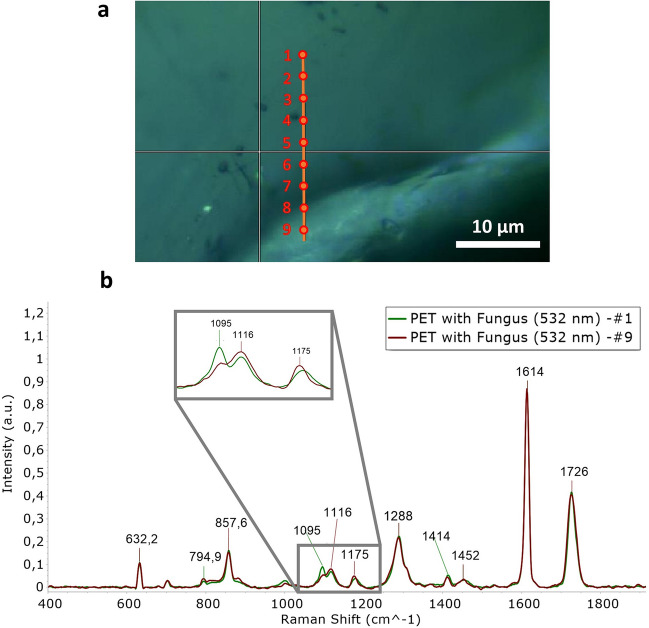
Raman spectra over a fracture on a PET fragment attacked by the fungal strain. (**a**) Spots analysed covering the fracture on the PET with fungus and (**b**) comparison between the spectra obtained from points 1 and 9.

Then, Raman analysis was performed on 9 points in the edge growth site in the vicinity of a fracture (Fig. 6a) in a colonised sample. Comparing the spectra obtained from points 1 to 9 a difference in 1080–1140 cm^–1^ band was observed (Fig. 6b). These results suggested conformation and crystallinity variation, of PET fragments in correspondence of the fracture^52^, that could be due to the packing of glycolic and trans-glycolic units in the polymer^53,54^ caused by the fungal action^55^. Indeed, changes in this region of Raman spectrum are usually attributed to carbonyl (C=O) stretching (1726 cm^–1^), or to a mixed modes of ring CH in-plane bending, glycol C–O stretching, COC and CCO bending, and C–C stretching of PET that are especially conferred to 1115 cm^−1^, 1094 cm^−1^, and 998 cm^−1^ peaks. Modifications in these regions are commonly used to evaluate the PET degradation^52^. The obtained results showed an increase in trans-glycolic units from the spectrum of point 1 to the spectrum of point 9 and a general weakening of the peak at 1095 cm^–1^ attributed to the ester bond^36^. Such a local variation in intensity (decrease in the intensity of the peak present at 1095 cm^–1^) near fractures could be attributed to the activity of cutinases enzymes, of which the strain F2 was a good producer, as shown in the preliminary enzyme assays. Indeed, cutinases are able to hydrolyse the scissile ester bonds in PET as well as they usually do during plants infection processes^33^.

These outcomes suggest that the presence of F2 induces structural modifications in PET encompassing regions where its concentration was more pronounced, notably in areas characterised by fractures along the edge. The absence of these changes in the PET reference samples confirms the action of the fungus. Thus, going back to Fig. 2 after these in-depth analyses, the light blue phase with geometric shapes right above the filaments could be salt crystals either from the culture Bushnell-Haas (BH) medium or produced by the fungus (Fig. 2c), the red phase with filaments formations recognized by XRM could be attributed to F2 fungal hyphae (Fig. 2d), and finally the yellow region surrounding the filaments inside the PET fragment could be identified as biodegraded PET (Fig. 2e), since the changes operated by the fungus altered the original density.

### Fungal penetration ability into PET

**Figure 7:**
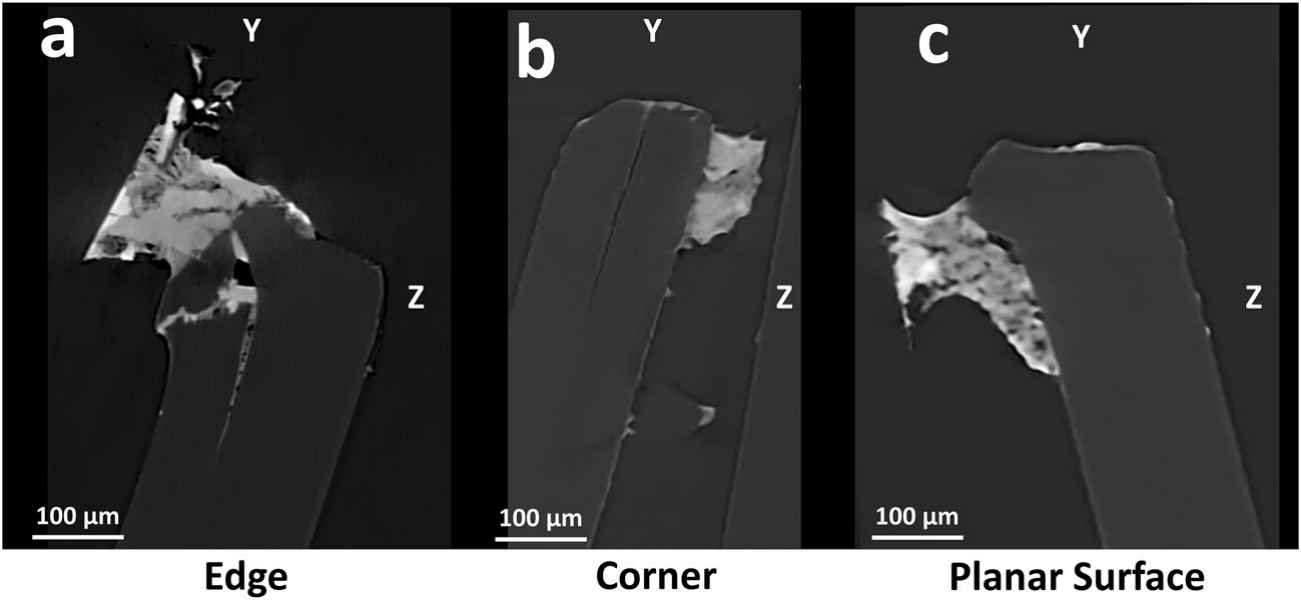
XRM virtual cross-sections of growth sites. The *Scout & Zoom* protocol coupled with the state-of-art *DeepScout* DL reconstruction algorithm was employed to identify smaller VOIs that correspond to the edge, corner, and planar surface fungal growth sites. These selected VOIs were rendered at higher resolution (pixel size 0.75 μm). After examining the 2D slices in the YZ plane, which depict internal cross-sections of PET fragments, it becomes evident that F2 *F. oxysporum* was able to penetrate deeply into PET near an edge (**a**), or, somewhat less extensively, near a corner (**b**). On the other hand, on the flat surface, there was only superficial growth (**c**).

The selected VOIs (edge, corner and planar surface of the sample) exhibited notable differences in terms of the hyphal penetration ability in relation to the PET substrate, when observed with XRM. Upon examining the 2D slices in the YZ plane, which represent interior cross-sections of PET fragments, it was noted that F2 managed to penetrate the PET substrate near an edge or a corner (Fig. 7a,b), while it remained confined on the surface of the planar portion of the fragment (Fig. 7c). Indeed, the fungal hyphae were able to penetrate deeply and develop inside the PET fragment, exploiting a probably pre-existing fracture present on the edge and enlarging it (maximum depth 250 μm, Fig. 7a). This behaviour was also observed at the PET corner growth site, although in a less pronounced way (Fig. 7b). On the other hand, the planar surface of PET was mostly unbroken, offering thus less chances for hyphal penetration and promoting only superficial growth (Fig. 7c). A similar approach to substrate was reported for the invasion of keratinous materials. Indeed, an initial surface erosion, from the outside toward the centre, is followed by radial penetration, when hyphae penetrate perpendicularly to the surface perforating the substrate to develop^56,57^. In presence of a keratinous substrate, *F. oxysporum* developed longitudinal broad perforators^58^. In plants, colonisation by a pathogen fungus usually starts from the penetration of the cuticle to reach and colonise the inner cells of the host. This process can occur thanks to the presence of stomatal pores and natural cracks, that allow the hyphal penetration^33^. The exploitation of pre-existing microcracks for the substrate penetration of hyphae was also found in volcanic rocks through XRM analysis^59^. Moreover, fungi can exploit mechanical pressure and hydrolytic enzymes, such as cutinase, to pierce the surface^33^. Based on the obtained VOIs results, both these penetration modes were employed during PET penetration.

**Figure 8:**
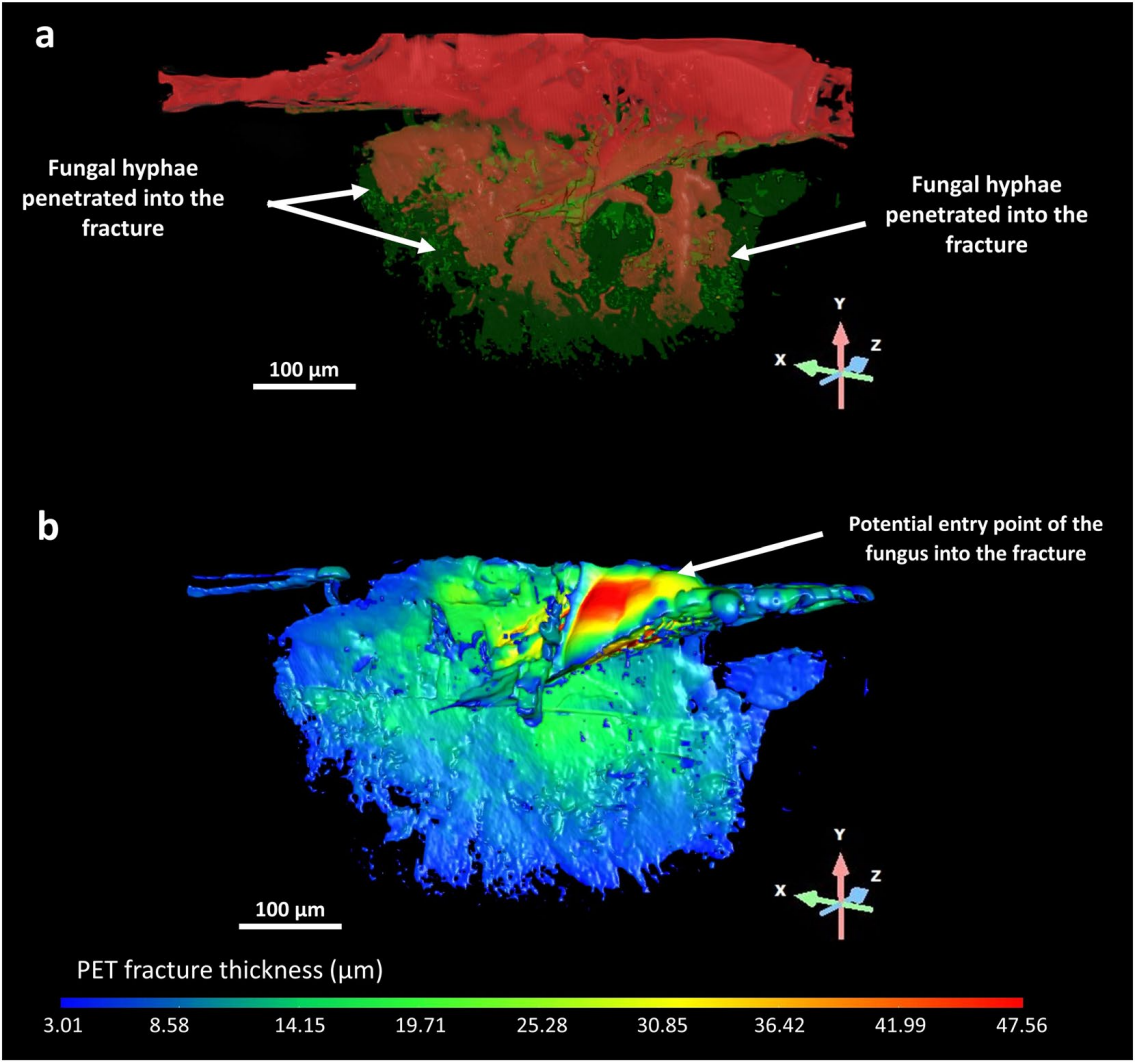
3D distribution and colonisation of F2 in PET fracture. The fracture previously highlighted in Fig. 7a was further investigated to get a better understanding of the three-dimensional distribution and colonisation pattern of the fungal strain within the fracture. (**a**) The surface fracture in PET is reported as a green mesh where the fungal strain, labelled in red, exhibited deep hyphal penetration, as indicated by the white arrows. (**b**) The fracture area was then converted to a thickness mesh. The peripheral area of the three-dimensional thickness colour-based map, depicted in thin blue, had a thickness ranging between 3 and 10 μm. It can be observed that this peripheral area has not yet been colonised by the fungus. The adjacent green region, corresponding to a width of 25 μm down to 10 μm, was formed by the growing fungal mycelium that increased the fragmentation of the substrate. The upper red area of the thickness map represents the wider zone (47.5 μm) of the fracture and could represent the access point of the fungus into the fracture.

The fracture present in Fig. 7a was investigated further to study the three-dimensional distribution and colonisation pattern of F2 within the fracture. To achieve this, the fracture surface was segmented using a histogram-based thresholding method and subsequently refined through a masking and a process islands workflow using Dragonfly Pro software. The resulting segmented surface was exported to a green mesh (Fig. 8a). The fungal strain, labelled in red, exhibited deep hyphal penetration, which is highlighted by the white arrows in Fig. 8a. The strain demonstrated a tendency to spread over the PET fracture surface and into the depth of the fracture. In order to obtain a three-dimensional thickness colour-based map of the fracture surface, this ROI was converted into a thickness mesh (Fig. 8b). This representation provided crucial insights into F2's ability to penetrate PET. The peripheral area of the three-dimensional thickness colour-based map (Fig. 8b), depicted in thin blue, had a thickness ranging between 3 and 10 μm. Notably, when comparing Fig. 8a and b, it can be observed that this peripheral area has not yet been colonised by the fungus. The adjacent green region, corresponding to a width of 25 μm down to 10 μm, was formed by the growing fungal mycelium, which, entering deeply in the fracture, increased the fragmentation of the substrate, leading to the possible degradation of this recalcitrant material. The upper red area of the thickness map represents the wider zone (47.5 μm) of the fracture which, based on the obtained XRM images, could represent the access point of the fungus into the fracture. This operational manner of penetration was also observed in *F. langsethiae* during colonisation of small grain cereals. Indeed, hyphae developed on the caryopsis edge and then formed focal points, specific sites where penetration took place. After the penetration, the infection hyphae branched from focal points inside the epidermis^60^. Similar behaviour was detected in *F. oxysporum* cabbage infection: a preliminary surface attachment was followed by the penetration and colonisation of the host. Thanks to confocal microscopy, it was clearly observed the hyphal penetration sites^61^.

## Conclusions

In this study we employed a multiscale correlative microscopy approach to investigate the infiltrative ability and degradative effects of the fungal strain F2, belonging to *F. oxysporum*, on PET fragments. The strain F2 was chosen for this study among the many isolated from soil with the aid of PET baits, as it was the one performing best in growth tests on PET and had an abundant production of the enzymes cutinases and esterases, which are heavily involved in plastic degradation*.* The use of non-destructive high-resolution X-ray Microscopy (XRM) coupled with the state-of-art Deep Learning (DL) reconstruction algorithm, called *DeepScout*, revealed that the fungal strain F2 exhibited a preferential distribution and enhanced penetration when growing in proximity to edges and corners of PET fragments, compared to planar surfaces. This observation can be attributed to a combination of physical, mechanical, and chemical factors that create microenvironments conducive to fungal growth. Indeed, the edges and corners of PET fragments exhibit a different topography compared to planar surfaces. Irregularities, such as crevices and sharp angles, may create microenvironments that are more conducive to fungal attachment and subsequent penetration and growth. The irregular surface topography may provide niches for fungal hyphae to establish and propagate. F2 may be attracted to these sites as they represent areas with increased accessibility and potential sources of nutrients. The fungal attack resulted in a multiphase region, with variations in morphology and composition. Indeed, this study allowed for the identification of three distinct phases constituting the sample, which, thanks to additional analyses with Raman spectroscopy and Energy Dispersive X-ray Spectroscopy (EDX), were identified as the fungal strain, biodegraded plastic, and salt crystals. The correlative microscopy approach enabled a comprehensive understanding of the interaction between the fungal strain and the PET substrate, shedding light on degradation processes, morphological changes, and potential structural alterations. This study highlights the importance of employing advanced microscopy techniques to investigate the complex dynamics of fungal plastic degradation, contributing to our understanding of potential strategies for mitigating plastic pollution. Further research in this field is warranted to explore the broader applicability of correlative microscopy in studying the interaction between fungi and recalcitrant materials. Future studies should focus on other fungal strains and substrates to elucidate the underlying mechanisms involved in the process. Additionally, the findings from these analyses could be correlated with more investigation tools to gain a deeper understanding of substrate biodegradation, including how its properties change during the process and in relation to fungal growth over time.

## Methods

### Fungal isolation and screening

The fungal strain used in this work was isolated from soil samples amended with polyethylene terephthalate (PET) baits. The soil used was collected from the Integral Nature Reserve Soil “Bosco Siro Negri”, located in a small strip of the Po Valley near the district of Pavia (Italy, PV, 45° 21′ 03.49″ N; 9° 05′ 78.16″ E; 65 m.a.s.l.). The fungal strains from this isolation campaign were subjected to growth tests on different plastic powders (PET, polyvinyl chloride (PVC), high density polyethylene (HDPE), polystyrene (PS), and polyurethane (PUR)), according to Temporiti et al.^62^. Moreover, the strains were screened for the production of the main enzymes involved in plastic degradation, such as laccases, lignocellulolytic enzymes, esterases, and cutinases. The qualitative enzyme assays for laccases, lignocellulolytic enzymes and esterases were performed according to Temporiti et al.^62^, while cutinases test was performed mixing NaNO_3_ (3 g/L), K_2_HPO_4_ (1 g/L), KCl (0.5 g/L), FeSO_4_.7H_2_O (0.01 g/L), 1% w/v flaxseed oil, phenol red (25 mg/L), and agar (17 g/L). These assays allowed the qualitative evaluation of the enzymatic activity intensity through the colour turning of the medium or particulate precipitation. From these tests, the best performing strain was selected for the multiscale correlative microscopy analysis in relation to its growth on PET. This strain, called F2, exhibited a high growth on PET and an abundant production of esterases and cutinases.

### Fungal identification

The strain F2 was initially identified by morpho-dimensional examination, based on the characteristics of the mycelium and of the reproductive structures. A molecular characterization was then performed to confirm and perfect the morphological identification. The fungal genomic DNA was extracted using the DNeasy UltraClean Microbial Kit (Qiagen, Hilden, Germany), following the manufacturer's instructions, and then subjected to PCR amplification of the ITS region of the ITS1-5.8S-ITS2 rDNA gene, using the primers ITS1 (5′-TCCGTAGG TGAACCTGCGG-3′) and ITS4 (5′ TCCTCCGCTTATTGATATGC-3′)^63^. The PCR reaction was performed according to the protocol by Daccò et al.^64^, changing only the annealing temperature to 55 °C. ExoSAP-IT (Applied Biosystems, Foster City, CA, USA) was used to purify the PCR according to the manufacturer’s instructions. The amplified and purified DNA was sent to BMR Genomics (Padova, Italy), and the sequences were compared with target sequences using BLAST online^65^ and MEGA X 10.1.7.

### Sample preparation

A fungal suspension of F2 was prepared by transferring the mycelium with a sterile needle from a 7-day-old potato dextrose agar (PDA) Petri dish into deionized sterile water (7 mL), and vortexing it. PET fragments (5 mm × 3 mm) were washed in sterile water and placed into sterile tubes, along with Bushnell-Haas medium (BH, 2 mL) and fungal suspension (200 µL). Reference samples were set up putting the PET fragments in BH medium without fungal suspension. Both reference and inoculated samples were incubated at 25 °C for 90 days.

### Scanning electron microscopy (SEM) analysis

Scanning electron microscopy (SEM) analysis was performed at KCS Biotech SRL (Vergiate (VA), Italy) and Arvedi Laboratory (University of Pavia, Italy) to visualise the placement of the fungus on the PET fragment. Microstructural characterization was performed employing a TESCAN, Mira 3 XMU (TESCAN, Brno, Cechia) operating at 8 kV and equipped with an In-Beam SE detector and a ZEISS Sigma 300VP (Carl Zeiss, Oberkochen, Germany) operating at 10 kV. Samples were previously coated with platinum (Pt) using a Cressington 208HR (Cressington Scientific Instruments, Watford, UK).

### X-ray microscopy (XRM) analysis

The initial survey of the sample was performed through low resolution XRM using a ZEISS Xradia Versa 610 (Carl Zeiss, Oberkochen, Germany) available at the Research Center on Nanotechnology Applied to Engineering (CNIS), Sapienza University of Rome to non-destructively collect three-dimensional data of samples. ZEISS Xradia Versa 610 microscope system provided the ability to non-destructively isolate a smaller region of interest (ROI) for higher-resolution XRM analysis thanks to its unique optics-based design. This procedure, also known as *Scout & Zoom,* was used to probe inner VOIs of samples using 4 × and 20 × objectives. Low-resolution XRM was performed with a pixel size of 4.33 μm, 4 × objective, camera binning 2, tube voltage 40 kV, source power 3 W, filter air, exposure time 4 s and acquiring 1601 projections. Absorption-contrast sub-micron XRM was performed setting a pixel size of 0.75 μm, 20 × objective, camera binning 2, tube voltage 80 kV, source power 10 W, filter air, exposure 3 s and acquiring 4,510 projections.

### Dataset reconstruction

The sets of projections were reconstructed using the Feldkamp-Davis-Kress (FDK) reconstruction algorithm and the state-of-art DL reconstruction algorithm called *DeepScout* (Carl Zeiss X-ray Microscopy, Dublin, CA, USA)^29^. The algorithm uses high-resolution XRM datasets as training data for lower resolution, larger FOV datasets and upscales the larger volume data using a neural network model. A selected high-resolution scan is used to train the model, enabling it to subsequently provide the higher resolution for the entire FOV.

### Image processing

The grey-scale level of a reconstructed XRM dataset refers to the intensity of a pixel in an image, represented by shades of grey. Each pixel’s grey-scale level corresponds to the amount of X-ray attenuation that occurred as the X-rays passed through the corresponding region of the sample. This allows a histogram thresholding segmentation process to label pixels and distinguish diverse phases, i.e. regions of interest (ROIs), that compose the sample. The segmentation process was carried out on reconstructed datasets, as well as the 3D rendering and analysis, using Dragonfly Pro (V. 2022.1 Build 1259, Object Research Systems, Montreal, Quebec, Canada)^66^.

### Energy dispersive X-ray (EDX) and Raman spectroscopy analysis

The identification of the biodegraded plastic was obtained using Energy Dispersive X-ray Spectroscopy (EDX) analysis, as well as Raman spectroscopy at the Research Center on Nanotechnology Applied to Engineering (CNIS), Sapienza University of Rome. Regarding the EDX parameters, experiments were performed using a Bruker Quantax mounted on a ZEISS Gemini Auriga (Carl Zeiss, Oberkochen, Germany) operating at 12 kV, WD 3.8 mm, MAG 3823x. Samples were previously coated with chromium (Cr) using a Quorum 250 T sputter coater**.** For the Raman analysis, a Renishaw InVia (Renishaw, Wotton-under-Edge, UK) was used. A PET reference sample, and a PET fragment where the fungus was grown were analysed through a static scan using a green laser (532 nm) with an output power of 5 mW, a long working distance (WD) 5 × objective (NA = 0.12), an exposure time of 1 s and 100 accumulations. The selected spectrum range was 400–1920 cm^–1^. Raman spectroscopy over fractures was performed for both samples using a short WD-100 × objective (NA = 0.85) in 1080–1140 cm^–1^ band.

## Data Availability

All relevant data are available from the corresponding author upon reasonable request.
